# The Immediate Effect of Trigger Point Injection With Local Anesthetic Affects the Subsequent Course of Pain in Myofascial Pain Syndrome in Patients With Incurable Cancer by Setting Expectations as a Mediator

**DOI:** 10.3389/fpsyt.2021.592776

**Published:** 2021-08-05

**Authors:** Hideaki Hasuo, Hiromichi Matsuoka, Yoshinobu Matsuda, Mikihiko Fukunaga

**Affiliations:** ^1^Department of Psychosomatic Medicine, Kansai Medical University, Hirakata, Japan; ^2^Department of Psycho-Oncology, National Cancer Center Hospital, Tokyo, Japan; ^3^Department of Psychosomatic Internal Medicine, National Hospital Organization Kinki-Chuo Chest Medical Center, Osaka, Japan

**Keywords:** immediate effect, expectation, pain reduction, myofascial pain syndrome, trigger point injection, local anesthetic, path analysis, indirect effect

## Abstract

A trigger point injection (TPI) with local anesthetic in myofascial pain syndrome (MPS) often has the immediate effect of a decrease in pain. It is unknown whether the immediate effect of a decrease in pain affects the subsequent course of pain. It is also unknown whether expectations of a decrease in pain mediate such effects. We aimed to clarify how the effect of a decrease in pain immediately after TPI with local anesthetic affected the subsequent course of pain, and whether it increased expectations of a decrease in pain. This was a prospective, single-center, observational clinical trial. Patients with incurable cancer who visited the palliative care department and received TPI with local anesthetic for MPS were prospectively examined. We evaluated whether the immediate effect of a TPI with local anesthetic affects the subsequent course of pain in MPS by setting expectations as a mediator, using path analysis. From 2018 to 2020, 205 patients with incurable cancer received TPI for MPS. Of these, 58.1% of patients reported an immediate effect of decreased pain. Compared with the non-immediate effect group, the immediate effect group had higher expectations of a decrease in pain, and the higher expectation was maintained at 7 days (*p* < 0.001). The percentage of patients with pain reduction at 7 days after TPI was 88.2% in the immediate effect group and 39.5% in the non-immediate effect group (*p* < 0.001). The immediate effect of decreased pain had the greatest influence on pain reduction at 7 days, both directly (β = 0.194) and indirectly through increased expectations (β = 0.293), as revealed by path analysis. The effect of a decrease in pain immediately after TPI with local anesthetic affected the subsequent course of MPS pain in patients with incurable cancer by setting expectations as a mediator. There were limitations to the discussion of these findings because this was an observational study.

## Introduction

The expectations of a decrease in pain have a favorable effect on the subsequent course of pain in patients. A systematic review suggested that positive expectations of a therapy is related to improved health outcomes (1). One study reported that positive pretreatment expectations of a decrease in pain have positive effects on the subsequent course of pain in cancer patients (2). Recovery expectations of a therapy also play an important role in placebo effects (3). It has been noted that placebo effects are independently and positively involved in the treatment of pain other than cancer pain, such as migraine (4).

To date, only a few studies have reported the factors that increase patients' expectations of a decrease in pain as a factor in placebo effects. Psychological, neurobiological, and genetic mechanisms have all been reported as factors affecting the placebo effect (5, 6). Furthermore, it has been proposed that placebo effects should be used to a maximum extent to raise patients' expectations and improve treatment outcomes (7). Psychological mechanisms may be more accessible in clinical practice compared with other factors. However, known psychological mechanisms that affect placebo effects are limited, although there are reports implicating the doctor–patient relationship and patients' beliefs about therapy (8, 9).

In clinical practice, one of the psychological mechanisms that increase patients' expectations of a decrease in pain is the immediate effect of a decrease in pain (an awareness of a feeling of comfort) immediately after treatment. Our group has previously reported that caregivers caring for family members with cancer are aware of a feeling of comfort immediately after a relaxation therapy session and that the feeling has positive effects on their future quality of life and autonomic nerve function by raising their expectations of the therapy (10). In the example of a decrease in pain, an immediate effect of a decrease in pain is frequently obtained with the use of anesthesia, alongside the inactivation of trigger points (TPs) immediately after trigger point injection (TPI) with local anesthetics. Between 31 and 45% of advanced cancer patients who complain of pain have TPs in their muscles, which are hypersensitive nociceptors (11, 12). However, while there have been reports of an association between pain and expectations, very few studies have investigated the immediate effect of a decrease in pain. It is unknown whether a decrease in pain immediately after TPI with local anesthetic has any effect on the subsequent course of pain in MPS by setting expectations as a mediator.

No standard treatment has yet been determined for patients with myofascial pain syndrome (MPS) (13), but TPI with local anesthetic is one of the most common treatments (14). MPS is a typical functional disease of pain. The diagnosis criteria established by Rivers et al. (15) indicate that TPs need to be palpated, and patients' pain needs to be recreated when pressure is applied to the TPs. Our group has previously reported that the efficacy rate of TPI in advanced cancer patients with MPS was 59% on the day after the injection (11). The aim of TPI is to reduce pain by inactivating TPs in the fascia of muscle. Common injectates include local anesthetics, botulinum toxin A, and steroids, but few controlled trials have been reported. One meta-analysis revealed that TPI with local anesthetic is more effective at mitigating pain intensity than botulinum toxin A (16).

We therefore hypothesized that the effect of a decrease in pain immediately after TPI would increase the expectations of a decrease in pain in MPS in cancer patients and that it would have a positive effect on the subsequent course of pain as a factor involved in the psychological mechanisms affecting placebo effects.

## Materials and Methods

### Study Design

This study was a prospective observational study that evaluated whether the immediate effect of TPI with local anesthetic affected the subsequent course of pain in MPS in patients with incurable cancer by setting expectations as a mediator.

### Ethics Statement

The study received approval from the Medical Ethics Committee of Kansai Medical University (reference number: 2018123). Informed consent was not obtained in this study because usual clinical practice was observed, including assessments and treatment. An opt-out method was used so that patients and their families could refuse to participate in the study. The procedures performed in this study were in accordance with the Declaration of Helsinki (as revised in 2013). This study was registered with the University Hospital Medical Information Network Clinical Trials Registry (approval number: UMIN000041210) on July 26, 2020 (retrospectively registered).

### Participants

This study was conducted from 2018 to 2020 at Kansai Medical University Hospital. During this period, patients with incurable cancer who visited the palliative care department were continuously enrolled. Among the enrolled patients, those who received TPI for MPS were included (inclusion criteria). MPS was diagnosed when the following criteria were met: (1) a tender spot was found with palpation, with or without referral of pain; (2) recognition of symptoms by the patient during palpation of the tender spot; and (3) at least three of the following: (a) muscle stiffness or spasm, (b) limited range of motion of an associated joint, (c) pain worsened with stress, and (d) palpation of a taut band and/or nodule associated with a tender spot (15). A diagnosis of MPS required careful manual examination, which considered that reliability estimates were generally higher for subjective signs such as tenderness and pain reproduction and lower for objective signs such as the taut band (17). Two exclusion criteria were applied to patients: those who (1) were younger than 20 years (because the invasive procedure required strict parental consent); and (2) had any comorbidity relating to psychiatric diseases or conditions that made communication difficult, such as cognitive impairment or delirium. The presence or absence of psychiatric diseases was evaluated by psychosomatic physicians using the Diagnostic and Statistical Manual of Mental Disorders, Fifth Edition.

### Intervention

TPI was applied by palliative care physicians to all MPS points observed as pain sites. For the TPI, 1 ml of 1% lidocaine was injected in an unblinded fashion, without ultrasound, to each pain site with a thin needle (27 G, 19 mm).

### Measures

#### Demographic Characteristics

Demographic information was obtained from all participants, and included age, sex, outpatients status, primary cancer site, medical treatments, Eastern Cooperative Oncology Group Performance Status (ECOG PS), site of MPS, number of MPS sites, analgesic drug use, and numerical rating scale (NRS) scores for pain and for the expectations of a decrease in pain (measured before TPI and immediately, 1, 3, and 7 days after TPI).

#### Measures of Pain Intensity and Criterion for Pain Reduction

The average pain intensity was assessed with an 11-point NRS for pain, ranging from 0 (no pain) to 10 (worst possible pain) (18). The questionnaire was self-administered and contained the following question: “How intense was your average pain over the past 24 h?” For multiple MPS sites, the average NRS score was used. The reliability and validity of this scale have been established previously (19). The criterion for pain reduction was determined as ≥33% improvement in the NRS score for pain before and after TPI (20).

#### Criterion for an Immediate Effect of a Decrease in Pain

The criterion for an “immediate” effect was determined as ≥33% improvement in the NRS score for pain before and immediately after TPI. The definition of immediate was between 5 and 20 min after TPI. The questionnaire contained the following question: “How intense is your current pain?”

#### Measures of Expectations of a Decrease in Pain and Criteria for Increased Expectations

The expectation intensity was determined with an NRS assessing expectations of a decrease in pain, ranging from 0 (no pain) to 10 (worst possible pain). The questionnaire was self-administered. The questionnaire contained the following question: “How much do you think your pain will decrease?” The criteria for increased expectations were determined as an NRS score ≥8, or ≥33% improvement in the NRS score assessing expectations of a decrease in pain before and immediately after TPI.

#### Outcomes

The primary outcome was a difference in the rate of patients with pain reduction at 7 days after TPI, between patients with and without a decrease in pain immediately after TPI. The secondary outcomes were changes in the NRS score for pain and in the NRS score assessing expectations of a decrease in pain, correlation and causality between the immediate effect, NRS score reduction at 7 days, increased expectations of a decrease in pain, and adverse events.

### Sample Size Calculation

The estimated number of patients required to detect a minimum rate of patients with a pain reduction difference of 0.15 (standard deviation, 0.10) between the two groups was 186. This value was estimated from published data (there are no data on the rate of patients with pain reduction at 7 days after TPI for patients with incurable cancer, so data from 1 day after TPI were used) (1). The significance was set at 0.05 and the power was set at 80%.

### Statistical Analysis

Data were reported as means with standard deviations, medians with interquartile range, or frequencies (%), as appropriate. The proportion of participants with an immediate effect of a decrease in pain among all participants and the rate of patients with pain reduction at 7 days after TPI for each group were estimated, including the exact 95% confidence intervals (95% CI).

The participants were classified into two groups: an immediate effect group and a non-immediate effect group. Unpaired *t*-tests were used among both groups for the dependent variables: age, number of MPS, NRS score for pain (day 0), and NRS score assessing expectations of a decrease in pain (day 0). Pearson's chi-squared tests were used to analyze the dependent variables: sex, outpatients, medical treatments, ECOG PS, site of MPS, and analgesic drug use.

Changes in the course of NRS scores for pain and NRS scores assessing expectations of a decrease in pain (day 0: before TPI and immediately after TPI, and on days 1, 3, and 7) were analyzed using one-way repeated measures analysis of variance (ANOVA) for each group. To conduct comparisons between groups, the time course was used as the within-participants factor and the group was used as the between-participants factor in two-way repeated measures ANOVA. In the ANOVA, multiple comparisons were corrected using the Bonferroni method. When a participant withdrew from the study, NRS scores after withdrawal were substituted with the score immediately before withdrawal. Changes in analgesic drug administration during the period and loss to follow-up were classified as withdrawal from the study.

Spearman's rank correlation coefficients were calculated to assess the associations between NRS reduction at 7 days after TPI and age, ECOG PS, site of MPS (lower back), number of MPS sites, analgesic drug use, NRS score (before TPI), expectations (before TPI), immediate effect of a decrease in pain, increased expectations (immediately and at 7 days after TPI), and NRS score reduction (7 days after TPI).

**Figure 1:**
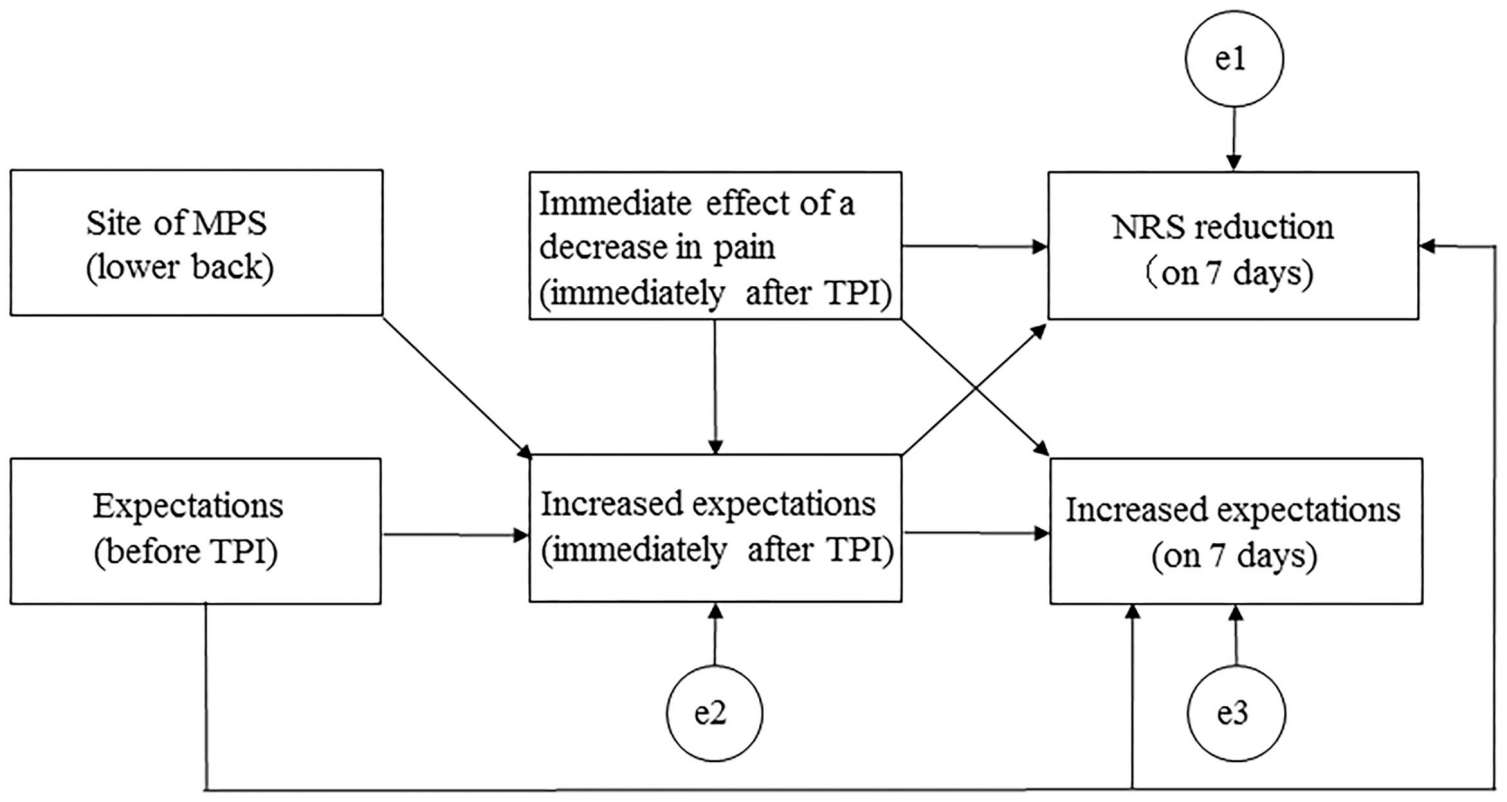
Path diagram for the hypothetical model. MPS, myofascial pain syndrome; NRS, numerical rating scale; TPI, trigger point injection.

Path analyses were conducted to estimate direct and indirect paths with reference to the aforementioned correlation coefficients. A hypothetical model was created in which expectations before TPI, a decrease in pain immediately after TPI, and increased expectations immediately after TPI predicted an NRS score reduction at 7 days. Expectations before TPI, a decrease in pain immediately after TPI, and the site of MPS (lower back) were mediators of increased expectations immediately after TPI and of an NRS score reduction at 7 days. Figure 1 shows the hypothetical model (Akaike information criterion [AIC] = 50.681). Path analyses were performed, deleting paths with *p* < 0.05 and adjusting parts with reference to the modification index, and repeating model correction while checking the goodness of fit (GFI) and investigating correlations between factors specifying an NRS score reduction at 7 days. To assess fit, model chi-squared values, GFI, comparative fit index (CFI), root mean square error of approximation (RMSEA), and AIC were used. For a good fit of the model, these measures should be as follows: chi-squared values from 2.0 to 5.0, GIF and CFI values > 0.95, and RMSEA values ≤ 0.08 (21). The AIC was used to compare the hypothetical model with the modified model, with a lower AIC indicating a better model.

A value of *p* < 0.05 was considered statistically significant. Statistical analyses were performed using SPSS version 25.0 and Amos version 25.0 for Macintosh (SPSS, Inc., IBM, Chicago, IL, USA).

## Results

### Number of Registered Patients

During the study period, 1,176 patients with incurable cancer who visited the palliative care department were continuously enrolled. Of the enrolled patients, MPS was observed in 394 patients with incurable cancer, and TPI was recommended in all cases. Of these 394 patients, 231 received TPI to the MPS site and 163 refused TPI for various reasons, including fear of injections. Of these 231 patients, 205 were selected as participants, after excluding 26 patients who met the exclusion criteria: (1) younger than 20 years (*n* = 3) and (2) any comorbidity relating to psychiatric diseases or conditions that made communication difficult (*n* = 23).

### Demographic Characteristics

Among all participants, 58.1% had an immediate effect of a decrease in pain (95% CI: 51.2–64.9). The participants were classified into the immediate effect group (*n* = 119) and the non-immediate effect group (*n* = 86). Table 1 shows the demographic and clinical characteristics of both groups.

**Table 1:** Clinical characteristics of the immediate effect group and the non-immediate effect group.

	**Immediate effect** **group (** ***n*** **=** **119)**		**Non-immediate** **effect group** **(** ***n*** **=** **86)**		***P*-value**
	**Mean**	**SD**	**Mean**	**SD**	
Age, years	68.3	11.7	66.5	12.7	0.318
	*n*	%	*n*	%	
Sex, female	58	48.7	32	37.2	0.117
Outpatients status	87	73.1	53	61.6	0.095
**Primary cancer site**					
Lung	11	9.2	4	4.7	
Gastrointestinal	40	33.6	37	43.0	
Liver, pancreas, biliary system	21	17.6	14	16.3	
Breast	15	12.6	7	8.1	
Gynecological	7	5.9	9	10.4	
Urological	4	3.4	6	7.0	
Head and neck	14	11.8	5	5.8	
Others	7	5.9	4	4.7	
**Medical treatments**					
Chemotherapy	76	63.9	55	64	1
BSC	43	36.1	31	36	
**ECOG PS**					
0–2	75	63.0	51	59.3	0.663
3–45	44	37.0	35	40.7	
**Site of MPS**					
Upper back	78	65.5	47	54.7	0.108
Lower back	39	32.8	39	45.3	
Others	2	1.7	0	0	
	**Mean**	**SD**	**Mean**	**SD**	
Number of MPS sites	2.8	1.2	2.5	1.3	0.140
**NRS for pain (Day 0)**					
Before TPI	5.9	2.4	6.3	2.4	0.239
Immediately after TPI	2.3	2.0	5.7	2.9	<0.001
**NRS assessing expectations for management of pain (Day)**					
Before TPI	4.9	2.4	5.1	2.4	0.537
Immediately after TPI	7.6	2.3	5.8	2.9	0.001
**Analgesic drug use**	***n***	**%**	***n***	**%**	
None	34	28.6	17	19.8	0.190
Use	85	71.4	69	80.2	
Non-opioid use	41	48.2	23	33.3	0.072
Opioid use	44	51.8	46	66.7	
	Median	IQR	Median	IQR	
Opioid dose (mg/day)^a^	30	20, 90	30	30, 55	

*BSC, best supportive care; ECOG, Eastern Cooperative Oncology Group; PS, performance status; MPS, myofascial pain syndrome; NRS, numerical rating scale; TPI, trigger point injection; SD, standard deviation; IQR, interquartile range*.

a *Dose of opioids is expressed as oral dose level of morphine (mg/dl). For conversion: parenteral morphine:oral morphine = 1:2, parenteral, oxycodone:oral morphine = 1:2, oral oxycodone:oral morphine = 2:3, fentanyl:morphine = 1:100, oral methadone:oral morphine = 1:8*.

Analgesic drug changes during the period were observed in 11 participants (5.4%). Four participants changed their analgesics because of worsened cancer-related pain in the immediate effect group. Seven participants changed their analgesics because of worsened cancer-related pain (*n* = 5) or self-interruption (*n* = 2) in the non-immediate effect group. No patient lost follow-up during the 7-day follow-up period.

### Between-Group Comparison of the Change Rate of NRS Scores

**Figure 2:**
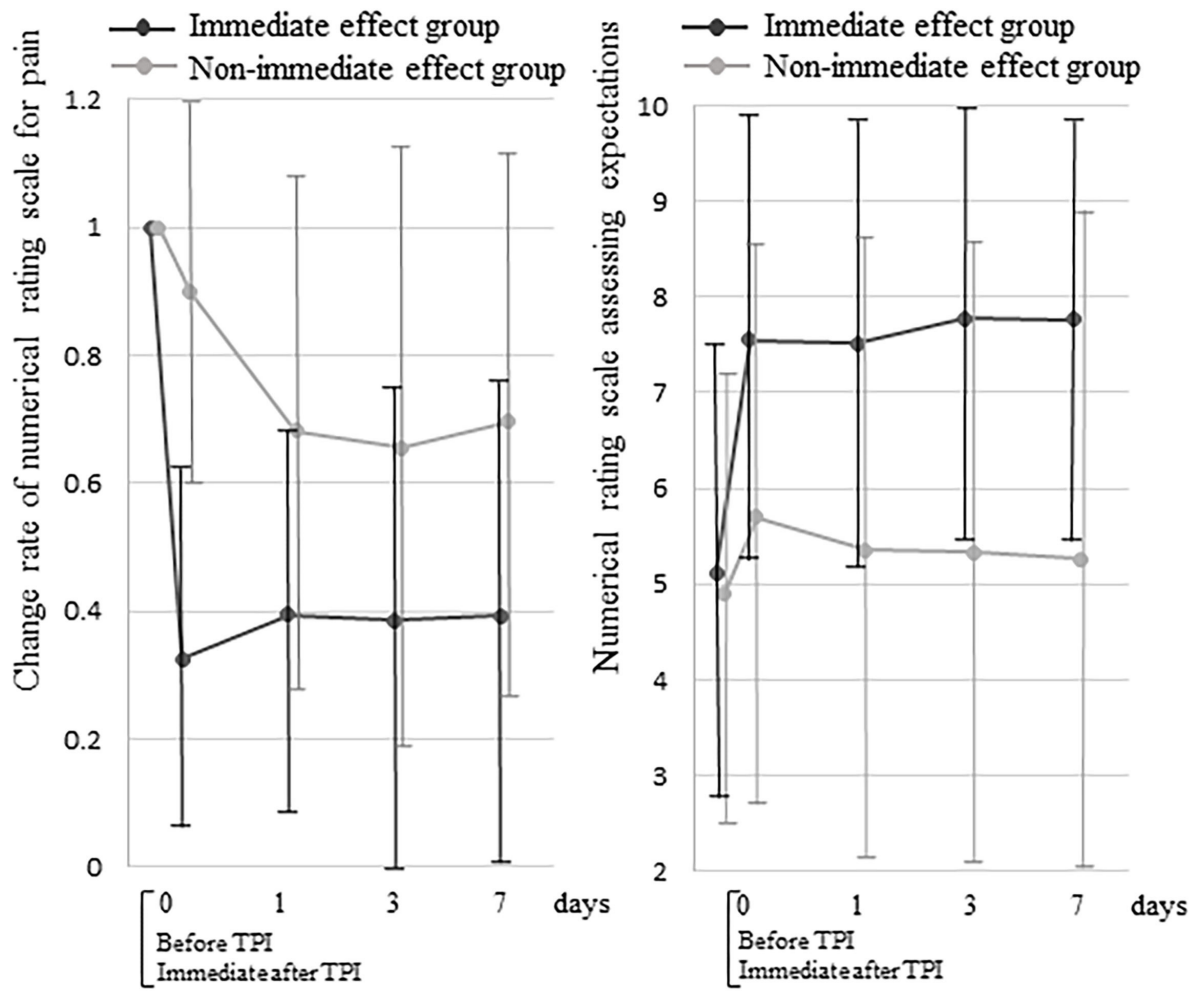
Between-group comparisons of the change rate of numerical rating scale scores for pain and the numerical rating scale scores assessing expectations of a decrease in pain. TPI, trigger point injection.

The NRS reduction rate at 7 days after TPI was 88.2% (95% CI: 82.4–94.1) in the immediate effect group and 39.5% (95% CI: 29.0–50.1) in the non-immediate effect group (*p* < 0.001) (Figure 2). The change rates of NRS scores in both groups were significantly lower at all times compared with the baseline (*p* < 0.001). Comparing the change rates of NRS scores between groups showed a significant interaction between the time course and group (*p* < 0.001). There was also a significant difference in time course between the two groups at day 0 (immediately after TPI) and on days 1, 3, and 7 (*p* < 0.001).

### Between-Group Comparison of NRS Scores Assessing Expectations

In both groups, the NRS scores assessing expectations of a decrease in pain were significantly higher at all times compared with the baseline (*p* < 0.001) (Figure 2). Comparing the NRS scores assessing expectations of a decrease in pain between groups showed a significant interaction between the time course and group (*p* < 0.001). There was also a significant difference in the time course between the groups at day 0 (immediately after TPI) and on days 1, 3, and 7 (*p* < 0.001).

### Correlation Coefficients Between NRS Score Reduction and Other Factors

The NRS score reduction at 7 days after TPI was correlated with expectations before TPI (correlation coefficient = 0.239, *p* = 0.001), the immediate effect of a decrease in pain (correlation coefficient = 0.514, *p* < 0.001), increased expectations immediately after TPI (correlation coefficient = 0.821, *p* < 0.001), and increased expectations at 7 days after TPI (correlation coefficient = 0.625, *p* < 0.001) (Table 2).

**Table 2:** Correlation coefficients between NRS score reduction at 7 days after trigger point injection and other factors.

	**1**	**2**	**3**	**4**	**5**	**6**	**7**	**8**	**9**	**10**	**11**
1. Age											
2. ECOG PS	0.046										
3. Site of MPS (lower back)	0.111	0.103									
4. Number of MPS sites	0.037	−0.048	−0.040								
5. Analgesic drug use	−0.160^*^	0.218^**^	−0.080	0.003							
6. NRS score (before TPI	−0.041	0.264^***^	−0.057	0.050	0.0146^*^						
7. Expectations (before TPI	−0.041	0.028	−0.046	−0.026	0.115	0.126					
8. Immediate effect of a decrease in pain	0.070	−0.127	−0.089	0.104	−0.100	−0.083	0.043				
9. Increased expectations (immediately after TPI	−0.012	−0.042	−0.139^*^	0.064	0.115	0.028	0.280^***^	0.421^***^			
10. Increased expectations (7 days after TPI	−0.014	0.032	−0.099	0.000	0.031	−0.060	0.183	0.339^***^	0.706^***^		
11. NRS reduction (7 days after TPI	0.061	−0.032	−0.094	0.05	−0.010	−0.109	0.239^**^	0.514^***^	0.821^***^	0.625^***^	

*ECOG, Eastern Cooperative Oncology Group; PS, performance status; MPS, myofascial pain syndrome; NRS, numerical rating scale; TPI, trigger point injection*.

* *p < 0.05*,

** *p < 0.01*,

*** *p < 0.001*.

### Path Diagram for the Final Model

**Figure 3:**
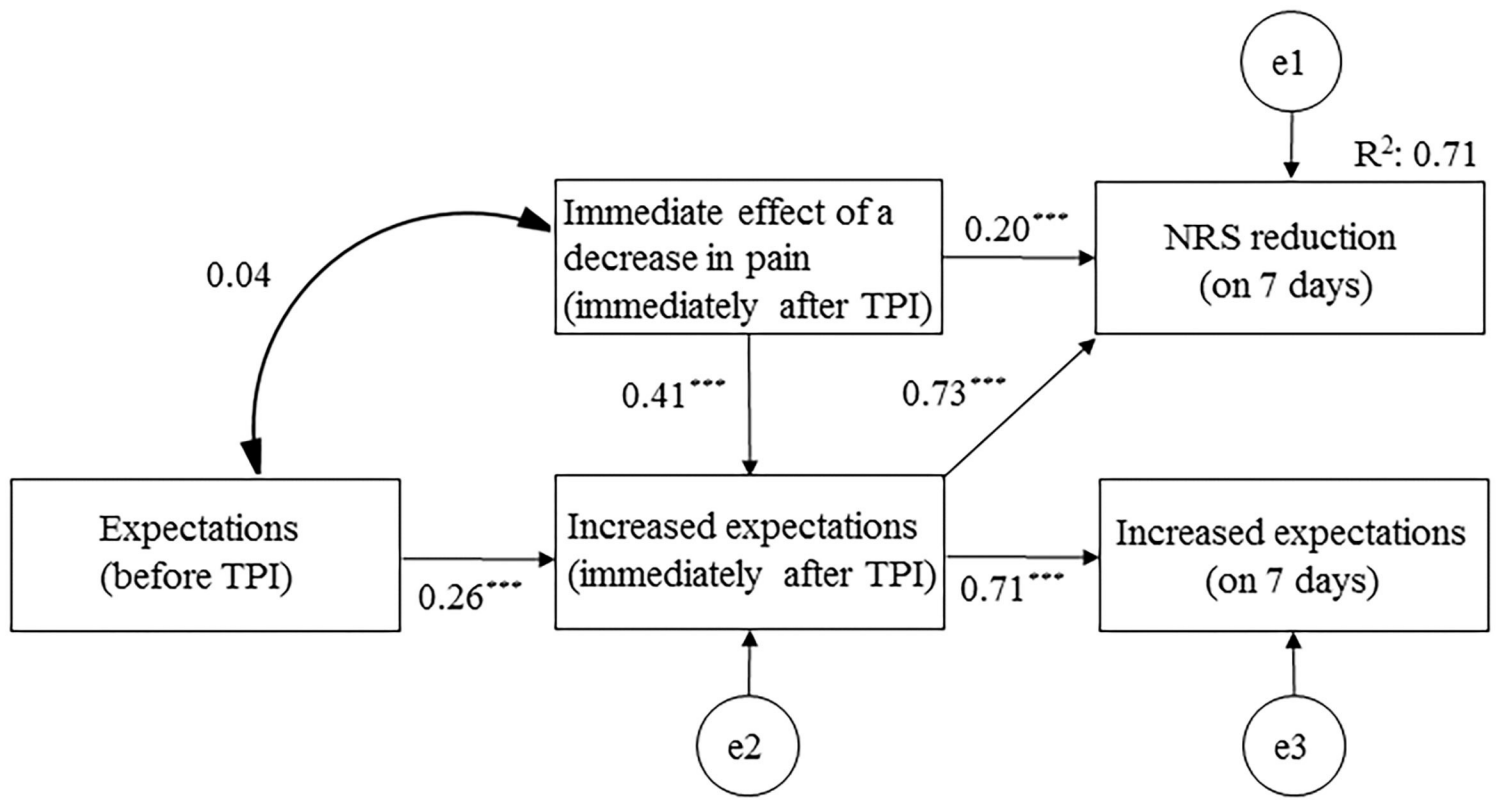
Path diagram for the final model. NRS, numerical rating scale; TPI, trigger point injection. ***p < 0.001.

The final model demonstrated the data well (model chi-squared value = 3.543, GFI = 0.993, RMSEA = 0.000, and AIC = 25.453). The immediate effect of a decrease in pain (immediately after TPI) had the most influence on NRS reduction at 7 days after TPI (β = 0.479) (Figure 3). The immediate effect of a decrease in pain affected NRS reduction at 7 days after TPI, not only directly (β = 0.194), but also indirectly through increased expectations (immediately after TPI) (β = 0.285). The overall fit of the model was examined using adjusted *R*^2^ values (0.708).

### Adverse Events

No adverse events occurred during any of the TPI performed throughout the evaluation period.

## Discussion

To the best of our knowledge, this study is the first to report the effect of a decrease in pain immediately after TPI on the subsequent course of pain and on increased expectations of a decrease in pain, *via* a study of MPS in patients with incurable cancer. However, there were limitations to the discussion of these findings because this was an observational study.

The first and most critical finding in the present study was that an immediate effect after TPI with local anesthetic indirectly affected pain reduction at 7 days after TPI, by setting increased expectations of a decrease in pain as a mediator. In the path analysis, the indirect effect (β = 0.285), with increased expectations immediately after TPI as a mediator, was larger than the direct effect (β = 0.194). Being aware of an immediate effect of a decrease in pain (a feeling of comfort) may lead to the expectation that subsequent pain reduction will continue. The immediate effect group had maintained a high NRS score assessing expectations of a decrease in pain by 7 days, so it was possible that longer follow-up effects of 7 days or longer could be expected. The effect of an immediate decrease in pain might have been one factor that increased patients' expectations of a future decrease in pain and may be involved in the psychological mechanisms of placebo analgesia. Fifteen percent of psychotherapy effects have been reported to be a placebo effect, and depend on patients' expectations for treatment (22). A previous study has demonstrated that being aware of a feeling of comfort immediately after a relaxation therapy has positive effects on future quality of life, by raising the individual's expectations of the therapy (10). Another study has reported that being aware of comfortable psychological and physiological changes immediately after hypnosis raised advanced cancer patients' expectations of the therapy and reduced symptoms of dizziness with an unknown cause (23).

The immediate effect of TPI with local anesthetic might be caused by its local anesthetic effect. Lidocaine is an aminoamide with an intermediate onset (10–15 min) and an intermediate duration of action (60–120 min), so it often does not have a local anesthetic effect the day after its use (24). The immediate effect group had a slight increase in pain the day after its use in the current study, probably because the local anesthetic effect had diminished by day 1 after TPI. In the present study, the increased expectations immediately after TPI were more affected by the immediate effects (correlation coefficient = 0.41) than the expectations before TPI (correlation coefficient = 0.26). Expectations of a decrease in pain after treatment were higher when expressed as an ideal expectation (81–93% relief) than as a predicted expectation (44–64%). In the current study, 67.4% of pain relief was observed as an immediate effect before and immediately after TPI. The immediate effects of a decrease in pain was a less-than-ideal expectation, but more than the predicted expectation. In TPI for MPS, the use of local anesthetics may maximize placebo analgesia *via* immediate effects.

The second important result in this study was that the immediate effect of TPI was associated with pain reduction at 7 days after TPI. The final path model revealed that 70.8% of the NRS score reduction after TPI to MPS sites in patients with incurable cancer could be explained by the immediate effect of a decrease in pain. This result may be useful when introducing treatments for cancer pain; for example, the ability to experience the immediate effects of rapid-release opioids may be tested before starting sustained-release opioids when introducing treatment with opioid analgesics.

The effect of a decrease in pain immediately after TPI might be a result of its inactivating effect of the TP. This might be one of the direct effects in path analysis, from the immediate effect to pain reduction at 7 days after TPI. The effect of TP inactivation by TPI might have occurred earlier in the immediate effect group than in the non-immediate effect group, but there are no previous reports on the timing (25). The shortest report of an onset of TPI effect is 1 day after TPI (11). In the present study, however, even the non-immediate effect group had a change rate in the NRS score for pain of ~33% or more from 1 day after TPI. A change of 33% is generally regarded as the minimal clinically important difference in pain outcome measures (20). Lidocaine is an aminoamide with an intermediate duration of action (60–120 min) and often does not have a local anesthetic effect the day after administration (24). This suggests that the onset of TP inactivation may occur 1 day after TPI.

### Limitations

The present study has several limitations. First, this study was an observational study, and there were therefore limitations to the discussion of these findings. However, this study provides a basis for future randomized controlled trials. Second, the NRS assessing expectations of a decrease in pain has been used frequently in previous research (2, 10), but the validity of this questionnaire is not clear. Third, only acute effects were evaluated, until 7 days after TPI treatment. This is because the treatment was targeted at patients with incurable cancer, so no long-term effects were assessed. We believed that immediate effects were more important for patients with incurable cancer who were close to death, even if they were short-term effects, and we selected patients with incurable cancer as the study participants. Fourth, we included patients with incurable cancer who visited our palliative care department, and it was difficult to generalize our results more widely in the cancer field. Finally, the study was conducted at a single facility with a lack of variation, and large-scale confirmation of the data is therefore warranted. Based on these limitations, the results of the current study should be considered to be preliminary.

## Conclusions

The effect of a decrease in pain immediately after TPI with local anesthetic affected the subsequent course of pain in MPS in patients with incurable cancer by setting expectations as a mediator.

## Data Availability Statement

The datasets presented in this study can be found in online repositories. The names of the repository/repositories and accession number(s) can be found in the article/supplementary material.

## Ethics Statement

The studies involving human participants were reviewed and approved by the ethics committee of Kansai Medical University, Japan (No. 2018123). Written informed consent for participation was not required for this study in accordance with the national legislation and the institutional requirements.

## Author Contributions

HH, HM, and YM were responsible for the conception and design of this study and data analysis. HH and MF were responsible for data collection and for clinical evaluations. HH wrote the article, which was critically revised by HM, YM, and MF. All authors have approved the final version of this manuscript.

## Conflict of Interest

The authors declare that the research was conducted in the absence of any commercial or financial relationships that could be construed as a potential conflict of interest.

## Publisher's Note

All claims expressed in this article are solely those of the authors and do not necessarily represent those of their affiliated organizations, or those of the publisher, the editors and the reviewers. Any product that may be evaluated in this article, or claim that may be made by its manufacturer, is not guaranteed or endorsed by the publisher.

## Abbreviations

TPI: trigger point injection
MPS: myofascial pain syndrome
TPs: trigger points
UMIN: University Hospital Medical Information Network
ECOG PS: Eastern Cooperative Oncology Group Performance Status
NRS: numerical rating scale
95% CI: 95% confidence intervals
ANOVA: analysis of variance
AIC: Akaike information criterion
GFI: goodness of fit index
CFI: comparative fit index.
